# Outcomes of hospitalizations with atrial fibrillation-flutter on a weekday versus weekend: an analysis from a 2014 nationwide inpatient sample

**DOI:** 10.7717/peerj.6211

**Published:** 2019-01-17

**Authors:** Dinesh C. Voruganti, Ghanshyam Palamaner Subash Shantha, Abhishek Deshmukh, Michael C. Giudici

**Affiliations:** 1 Division of Internal Medicine, Roy and Lucille J. Carver College of Medicine, University of Iowa Hospitals and Clinics, Iowa City, IA, United States of America; 2 Division of Cardiovascular Medicine, University of Michigan - Ann Arbor, Ann Arbor, MI, United States of America; 3 Division of Cardiovascular Medicine, Mayo Clinic, Rochester, MN, United States of America; 4 Division of Cardiovascular Medicine, Roy and Lucille J. Carver College of Medicine, University of Iowa Hospitals and Clinics, Iowa City, IA, United States of America

**Keywords:** Atrial fibrillation, Weekend, Cardioversion, Anticoagulation, In-hospital mortality, Time to cardioversion

## Abstract

**Background:**

Patients with atrial fibrillation-flutter (AF) admitted on the weekends were initially reported to have poor outcomes. The primary purpose of this study is to re-evaluate the outcomes for weekend versus weekday AF hospitalization using the 2014 Nationwide Inpatient Sample (NIS).

**Methods:**

Included hospitalizations were aged above 18 years. The hospitalizations with AF were identified using the international classification of diseases 9 (ICD-9) codes (427.31, 427.32). In-hospital mortality, length of stay (LOS), other co-morbidities, cardioversion procedures, and time to cardioversion were recorded. All analysis was performed using SAS 9.4 statistical software (Cary, North Carolina).

**Results:**

A total of 453,505 hospitalizations with atrial fibrillation and flutter as primary discharge diagnosis were identified. Among the total hospitalizations with a primary diagnosis of AF, 20.3% were admitted on the weekend. Among the weekend hospitalizations, 0.19% died in hospital compared to 0.74% among those admitted during the week. After adjusting for patient characteristics, hospital characteristics and disease severity, the adjusted odds for in-hospital mortality were not significantly different for weekend vs. weekday hospitalizations (OR = 0.91, 95% CI [0.77–1.11]; *p* = 0.33). The weekend admissions were associated with significantly lower odds of cardioversion procedures (OR = 0.72, 95% CI [0.69–0.76], *P* < 0.0001), lower cost of hospitalization (USD 8265.8 on weekends vs. USD 8966.5 on the weekdays, *P* < 0.001), slightly lower rate of anticoagulation (17.09% on the weekends vs. 18.73% on the weekdays. *P* < 0.0001), and slightly increased time to cardioversion (1.94 days on the weekend vs. 1.73 days on weekdays, *P* < 0.0005). The mean length of hospital stay (LOS) was statistically not different in both groups: (3.49 days ± 3.70 (SD) in the weekend group vs. 3.47 days ± 3.50 (SD) in the weekday group, *P* = 0.42)

**Discussion:**

The weekend AF hospitalizations did not have a clinically significant difference in mortality and LOS compared to those admitted on a weekday. However, the use of cardioversion procedures and cost of hospitalization was significantly lower on the weekends.

## Introduction

Atrial fibrillation is the most common persistent cardiac arrhythmia in clinical practice which had an estimated worldwide prevalence of 33.5 million in 2010 (Markides & Schilling, 2003). Patients with atrial fibrillation-flutter (AF) hospitalized on weekends were previously reported to have higher mortality and increased the length of hospital stay (Deshmukh et al., 2012; Shawn Lee et al., 0000). The factors thought to be contributing to the poor outcomes are: limited availability of the staff and access to procedures such as cardioversion. Similar studies on acute myocardial infarction have demonstrated higher inpatient mortality for weekend hospitalizations (De Cordova et al., 2017). A similar analysis on AF weekend hospitalizations reported improved mortality, (Weeda et al., 2016) but the improvement in these outcomes were consistently not replicated by studies performed on a large database on inpatient hospitalizations. We sought to investigate the outcomes in the year 2014 through publically available nationwide inpatient sample database (NIS) to assess the outcomes (in-hospital mortality, rates of cardioversion and time to cardioversion) and to determine whether such differences resulted from the disparities in the utilization and timing of cardioversion.

## Methods

The NIS is a part of the Healthcare Cost and Utilization Project (HCUP) which is sponsored by the Agency for Healthcare Research and Quality (AHRQ) (HCUP, 2012). Each year of the NIS records over 7 million inpatient hospitalizations. The NIS is one of the largest all-payer databases of hospital inpatient stays available in the United States of America (USA). The 2014 (1st January to 31st December) NIS sampling frame is comprised of 44 States and the District of Columbia, covering more than 96 percent of the U.S.A population and including more than 94 percent of discharges from the USA community hospitals.

Our main interest group was the hospitalizations who had a primary diagnosis (dx1) of atrial fibrillation or flutter. All hospitalizations with international classification of diseases, 9th revision, code 427.31 or 427.32 as the principal diagnosis were included. Per AHRQ-HCUP, the weekend admissions were defined as admissions on Saturday–Sunday (Ananthakrishnan, McGinley & Saeian, 2009). The use of cardioversion is denoted by the presence of ≥1 of the following ICD 9 procedural codes in any position: 99.61, 99.62 and 99.69. Our primary outcome was in-hospital mortality, and the secondary outcomes included utilization of cardioversion procedures, length of hospital stay (LOS), time to cardioversion, anticoagulation and total hospitalization charges.

The study protocol was reviewed by the University of Iowa, Iowa City, Institutional Review Board (IRB) (review: 201806023), and the study was exempt from human subject research as it includes only de-identified, publically available data. All analyses were performed using SAS, version 9.4 (SAS Institute, Cary, North Carolina). Survey procedures available within the SAS were applied in the analysis to account for design features of the complex sample survey. Descriptive statistics were generated for the individual and hospital characteristics for both weekend and weekday admissions. Univariate tests were applied to compare the equality of the mean or proportions for the motioned outcomes between the weekday and weekend admissions, which consisted of the Rao-Scott chi-square test for categorical outcomes and *t*-tests (along with standard deviation (SD)) for continuous outcomes. The cost of hospitalization was calculated from cost to charge ratio files and the total charges provided by the AHRQ (https://www.hcup-us.ahrq.gov/db/state/costtocharge.jsp). The number of chronic conditions was obtained from ‘NCHRONIC’ variable listed in the NIS database. The data element ‘NCHRONIC’ contains the count of unique chronic diagnoses reported on the discharge. The long-term (current) use of anticoagulants was determined using the ICD-9 CM code ‘V58.61’. The national estimates for hospitalization were calculated by applying the weights provided by the HCUP-AHRQ in the NIS file. Finally, multivariate logistic regression models were applied to test the adjusted associations between the outcomes of weekend versus weekday admissions. The level of significance (*α*) was chosen as 5%.

## Results

We identified a national estimate of 453,505 hospitalizations with AF as the primary diagnosis. Of these, 92,220 were characterized as weekend hospitalizations and 361,285 as weekday hospitalizations. The mean age among weekend and weekday was statistically not different (weekday 70.1 years ± 13.5 (SD) and weekend 70.2 years ± 14.2 (SD)) with a *P*-value of 0.53 (*T*-test). The proportion of males was somewhat lower in the weekend group (48.13% on weekends vs. 50.53% on the weekdays, *P* value < 0.0001). A slightly lower proportion of the white population was hospitalized over the weekends (80.31% on the weekends vs. 82.60% on the weekday), and a higher proportion of the Hispanic population was admitted on the weekends (6.25 on weekends vs. 5.34 on the weekdays, *P* < 0.0001). Hospitalizations with Medicare constituted the majority of overall hospitalizations (67.13%) for AF. AF hospitalizations were relatively higher in the Urban teaching hospitals (59.61%), and the weekday hospitalizations were higher in the urban teaching hospitals vs. the weekend (60.12 on the weekday vs. 57.63% on the weekends, *P* < 0.0001). The same pattern was observed in the large hospitals which constituted about 50.64% of total AF hospitalizations, and a slightly higher rate of AF hospitalizations in the large hospitals was on the weekdays (51.08% on the weekday vs. 48.93% on the weekends, *P* < 0.0001). Table 1 summarizes the demographics and baseline characteristics for the weekday and weekend hospitalizations along with the *P* values (*T*-test for continuous variables and Chi-square test for the categorical variables).

**Table 1:** Baseline characteristics of atrial fibrillation (AF) hospitalizations. The baseline characteristics indicate the percentage of AF hospitalizations admitted on the weekends and the weekdays. The total hospitalizations include both groups. The *P* value indicates the chi-square test for the baseline characteristic differences among the weekday and the weekend groups.

Characteristic	Weekday hospitalization (*n*= 361,285)	Weekend hospitalizations (*n*= 92,220)	Total hospitalizations (*n*= 453,505)	*P* Value
Mean age (years) ± (Standard deviation)	70.1 ± 13.5	70.2 ± 14.2	70.1 ± 13.6	0.5338
Gender				<0.0001
Male	50.53%	48.13%	50.0%	
Female	49.46%	51.86%	49.95%	
Race				<0.0001
White	82.6%	80.31%	82.1%	
Black	8.18%	9.03%	8.36%	
Hispanic	5.34%	6.25%	5.53%	
Asian	1.36%	1.66%	1.42%	
Native American	0.38%	0.43%	0.39%	
Other	2.10%	2.30%	2.14%	
Primary Payer				<0.0001
Medicare	66.95%	67.83%	67.13%	
Medicaid	5.84%	6.41%	5.96%	
Private	22.61%	20.63%	22.21%	
Self-pay	2.43%	2.94%	2.54%	
No charge	0.27%	0.38%	0.29%	
Other	1.86%	1.78%	1.84%	
Hospital region				<0.0001
Northeast	21.10%	20.07%	20.89%	
Midwest	24.55%	23.65%	24.37%	
South	39.71%	40.34%	39.84%	
West	14.62%	15.92%	14.89%	
Type of admission				<0.0001
Elective	14.34%	4.14%	12.26%	
Non-Elective	85.65%	95.85%	87.73%	
Hospital location/teaching status			11.32%	<0.0001
Rural	11.22%	11.72%	29.05%	
Urban non-teaching	28.65%	30.63%	59.61%	
Urban teaching	60.12%	57.63%		
Hospital bed size				<0.0001
Small	18.88%	19.60%	19.03%	
Medium	30.02%	31.45%	30.31%	
Large	51.08%	48.93%	50.64%	
CHA_2_DS_2_VASc score (mean ± standard deviation)	2.73 ± 1.44	2.79 ± 1.47	2.74 ± 1.45	<0.0001

Comparing the in-hospital mortality (primary outcome) in two groups, we have identified that the mortality for weekend hospitalizations did not significantly vary from the weekday hospitalizations (0.19% on the weekends vs. 0.74% on the weekdays, *P* = 0.90).

Secondary outcomes were the number of inpatient cardioversion procedures, interval to the procedure (time to cardioversion), length of stay, anticoagulation and the cost of hospitalization. These characteristics are listed in Table 2. We noted that the weekend AF hospitalizations underwent fewer cardioversion procedures than those hospitalized on a weekday (2.90% vs. 14.83%, *p* < 0.0001). The average time to cardioversion was not very different among both groups, though statistically significant (1.94 days on the weekend vs. 1.73 days on a weekday, *P* = 0.0005). The weekend AF admission was associated with a lower cost of hospitalization (USD 8265.8 on weekends vs. USD 8966.5 on the weekdays, *P* < 0.001). The weekend hospitalizations had a slightly lower rate of anticoagulation (17.09% on the weekends vs. 18.73% on the weekdays. *P* < 0.0001).

**Table 2:** Differences between outcomes for the weekday and weekend hospitalizations for Atrial Fibrillation (AF). The differences in the the weekday and the weekend hospitalizations indicates the percentage of hospitalizations for AF. The *P* value indicates the differences in these groups after performing the chi-square and *t*-test.

Variable	Weekend admission (*n*= 92,220)	Weekday admission (*n*= 361,285)	*P*-value
Cardioversion	2.90%	14.83%	*P* < 0.0001
Mean length of stay (days) ± Standard deviation	3.49 ± 3.70	3.47 ± 3.50	*P* = 0.4233
In-hospital mortality	0.19%	0.74%	*P* = 0.9058
Mean cost of hospitalization (USD)	8265.8	8966.5	*P* < 0.001
Time to cardioversion (days) ± (standard deviation)	1.94 ± 2.40	1.73 ± 3.96	*P* = 0.0005
Anticoagulation	17.09%	18.73%	<0.0001

**Figure 1:**
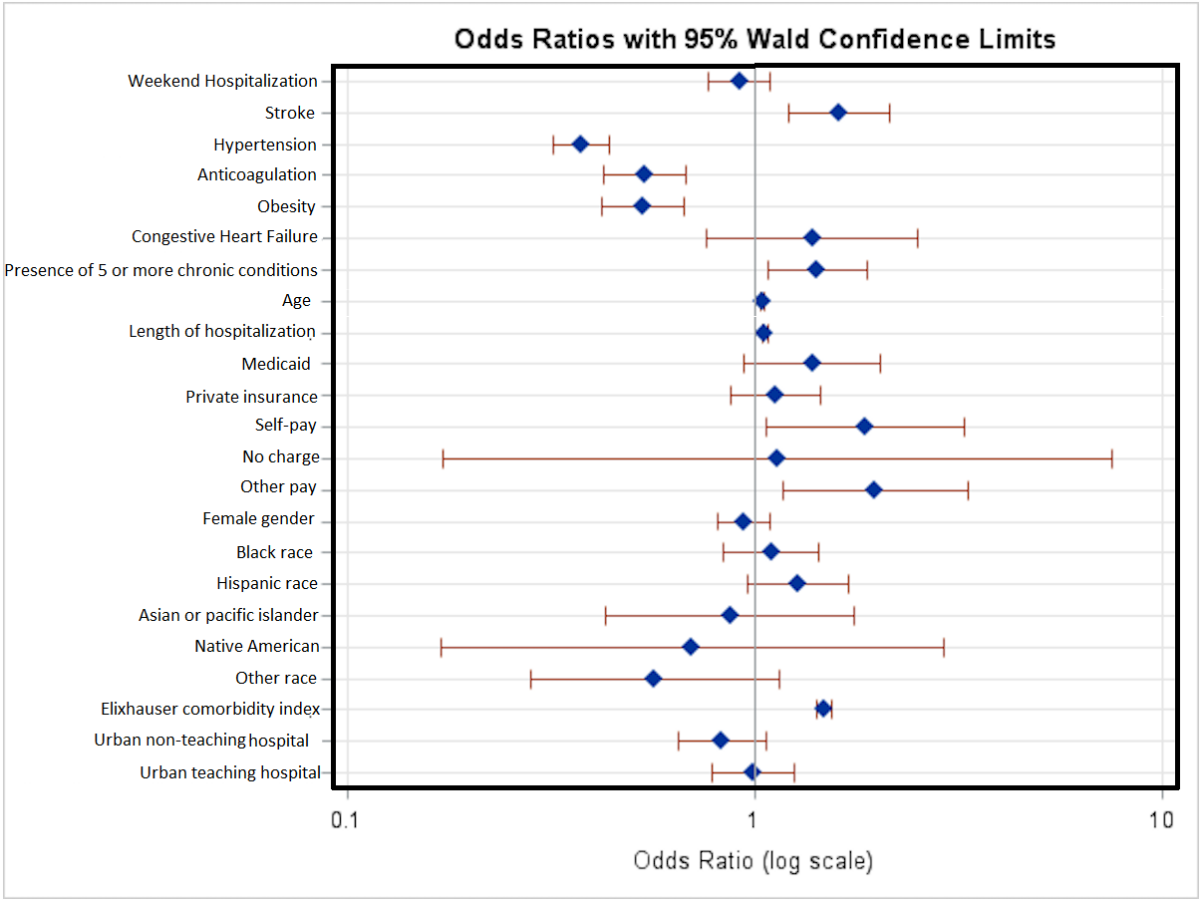
Multivariate logistic regression analysis with adjusted odds ratio’s for in-hospital mortality. The adjusted odds ratios, 95% confidence intervals and their *P*-values represent the odds of in-hospital mortality after adjusting for the covariates listed in the table. The blue dots indicate the adjusted odds ratio for the listed variable, and the red lines indicate 95% confidence intervals. OR to the right of midline (where OR = 1) indicate higher odds of in-hospital mortality while OR to the left of the midline indicate lower odds of in-hospital mortality.

The univariate (Table 3) and the multivariate logistic regression analysis for in-hospital mortality were performed, after adjusting for significant covariates such as age, sex, hypertension, obesity, diabetes mellitus, congestive heart failure, stroke, anticoagulation, length of stay (LOS), primary expected payer, race, hospital location and the teaching status. We observed that the weekend hospitalizations did not have significant difference for in-hospital mortality OR = 0.917 (95% CI [0.77–1.092]; *P* = 0.3299) (described in Table 4, Fig. 1). Also, we observed that the hospitalizations with a diagnosis of stroke and presence of 5 or more chronic conditions had the most significant association with in-hospital mortality OR= 1.609 (95% CI [1.214–2.132], *P* = 0.0009) and OR = 1.423 (95% CI [1.074–1.886], *P* = 0.014) respectively.

**Table 3:** Unadjusted estimates for in-hospital mortality for AF hospitalizations. The unadjusted odds ratios indicate the univariate association between the comorbidity listed in the first column to the in-hospital mortality. This indicates the strength of association without adjusting for other variables.

**Unadjusted odds ratio’s to in-hospital mortality**				
	OR	95% Confidence interval		*P*-value
Stroke	2.67	2.07	3.45	<0.0001
Hypertension	0.73	0.63	0.85	<0.0001
Anticoagulation	0.52	0.42	0.66	<0.0001
Obesity	0.59	0.48	0.73	<0.0001
Congestive Heart Failure	8.27	5.14	13.32	<0.0001
≥5 Chronic Conditions	3.12	2.44	3.98	<0.0001
Weekend admission	1.01	0.85	1.19	0.0140
Female	1.24	1.09	1.42	0.0011

**Table 4:** Multivariate logistic regression analysis showing the adjusted odds ratio’s predicting the in-hospital mortality for Atrial Fibrillation (AF) hospitalizations. The adjusted odds ratio’s, 95% confidence intervals and their *P*-values represent the odds of in-hospital mortality after adjusting for the covariates listed in the table.

**Odds ratio estimates**				
**Effect**	**Adjusted odds ratio**	**95% confidence limits**		***P*-value**
Weekend hospitalization	0.917	0.77	1.092	0.3299
Stroke	1.609	1.214	2.132	0.0009
Hypertension	0.376	0.32	0.441	<.0001
Anticoagulation	0.538	0.427	0.679	<.0001
Obesity	0.529	0.42	0.667	<.0001
Congestive Heart Failure	1.383	0.76	2.516	0.2886
≥5 Chronic Conditions	1.423	1.074	1.886	0.014
AGE	1.048	1.039	1.057	<.0001
Length of stay	1.059	1.043	1.076	<.0001
EXPECTED PRIMARY PAYER				
Medicare (Reference group)				
Medicaid	1.385	0.941	2.039	0.0984
Private insurance	1.124	0.873	1.448	0.3643
Self-pay	1.868	1.065	3.275	0.0292
No charge	1.14	0.172	7.552	0.8918
Other pay	1.978	1.175	3.329	0.0103
Female gender	0.936	0.807	1.086	0.3814
RACE				
White (Reference group)				
Black	1.099	0.837	1.441	0.4971
Hispanic	1.273	0.956	1.695	0.0986
Asian or pacific islander	0.869	0.43	1.757	0.6962
Native American	0.702	0.169	2.911	0.6259
Other	0.569	0.282	1.146	0.1144
Elixhauser comorbidity index	1.474	1.412	1.538	<.0001
HOSPITAL LOCATION AND TEACHING STATUS				
Rural hospital (Reference group)				
Urban non-teaching hospital	0.832	0.651	1.064	0.143
Urban teaching hospital	0.99	0.788	1.244	0.933

## Discussion

The main inferences of our analysis on the NIS 2014 data are: (1) AF weekend hospitalizations showed no clinically significant differences in mortality, length of stay, time to cardioversion; (2) we also found that weekend hospitalizations were less likely to undergo cardioversion, and (3) they had overall lower mean cost of hospitalization.

Prior study on AF weekend hospitalization by Deshmukh et al. (2012) reported the adjusted in-hospital mortality to be higher for weekend admissions (OR 1.23, CI [1.03–1.51]), longer weekend length of hospitalization and lower rates of utilization of cardioversion (7.92% weekend vs. 16.2% weekday). Similarly, another study by Weeda et al. (2016) has reported that there were no differences in the adjusted in-hospital mortality rate (OR 1.02; 95% CI [0.94–1.11]) and the length of hospitalization. However, they were found to have longer time-to-procedure and lesser treatment costs with weekend admissions.

In comparison to the prior studies, our results match Deshmukh et al. (2012) where the utilization of cardioversion and cost of hospitalization was lower in weekend AF hospitalizations. On the other hand, our analysis also matches Weeda et al. (2016) in demonstrating a lack of significant difference in mortality and the length of hospitalization stay among both groups comparing weekend and weekday admissions. The outcomes on the weekend are informally referred to as the ‘weekend effect’. It is a phenomenon which is often highlighted to associate poor outcomes in weekend hospitalizations. It has been proposed that it might be a result of lack of healthcare management organizations to improve practices of care, which includes ensuring round the clock accessibility to life-saving procedures (Mathew et al., 2018). Also, published meta-analysis has associated poor outcomes in patients admitted with myocardial infarction and other medical conditions (Sorita et al., 2014). In patients with AF, the higher mortality, length of stay and lower utilization of cardioversion procedures were thought to be secondary to limited availability of services on the weekends. It was proposed that the subtle primary signs of acute problems go unnoticed until later on the weekends. Studies focused on the weekend effects, in general, have emphasized for a better-organized model of care which could help in bridging the gap of the weekend effect.

We notice an improvement of outcomes in hospitalizations with AF. The difference of in-hospital mortality, length of hospitalization, and time to cardioversion has been gradually decreasing since Deshmukh et al.’s (2012) publication. These changes might occur due to the implementation of robust patient care across the hospitals in the United States to provide 24/7 accessibility to procedures such as cardioversion and prompt recognition of subtle clinical parameters such as atrial fibrillation with rapid ventricular response with improvised computerized telemetry, resulting in early transfer to the intensive care unit for cardioversion on the weekends (Albright et al., 2009; Conway et al., 2018). However, utilization rates of cardioversion continue to be low among the weekend AF hospitalizations. One of the reasons might be due to limited staff availability and sometimes, delay in identifying subtle signs of acute problems, such as hypotension, may go unnoticed until later.

On the other hand, the lack of mortality difference on the weekends with a lower cost of hospitalization and lower rates of utilization of cardioversion procedures also raise a concern about the higher costs of hospitalization on the weekdays. We notice a higher rate of cardioversion procedures on the weekdays which might also sometimes imply overutilization of the procedures, resulting in a higher cost of hospitalization. While opportunities to improve care on the weekends are constantly being explored, a cost-effective management strategy may also be pursued to reduce the costs of hospitalizations on the weekdays. In our analysis, we noted low anticoagulation rates in both the weekend and the weekday groups, which is in concordance with the report from the ‘Get with the Guidelines’, registry that showed similarly low rates (15% to 17%) of anticoagulation in their AF patients (Piccini et al., 2016). The low anticoagulation rates are probably because of high bleeding risk, higher prevalence of contraindications to anticoagulation in AF patients, or due to the coding inconsistencies.

Future directions should be focused on improving the utilization rates of cardioversion procedures and assessing the reasons for the disparity between the hospitalization costs associated with weekend hospitalizations. Our study findings provide valuable data demonstrating the improved mortality outcomes and length of hospitalization. Understanding the reasons behind the decreased cost of admission on weekends and reduced utilization of cardioversion procedures might help to bridge the gap difference.

Though our study had essential strengths of including a large sample, our study is subject to some limitations. First, the NIS relies on claims data which can incur inaccurate billing and underestimation of covariates of interest, thus leading to coding bias (Yoshihara & Yoneoka, 2014). Missing values in our data prevented us from including specific variables in the multivariate analysis. Furthermore, we did not evaluate the causes that could have accounted for this difference that are not patient related but related to the hospital (e.g., staffing differences on weekends).

## Conclusion

In the nationwide US practice, the weekend AF hospitalizations appear to have lower rates of cardioversion utilization and lower hospitalization cost. Further studies are required to identify the differences and explore the opportunities to improve AF weekend care.

##  Supplemental Information

10.7717/peerj.6211/supp-1 Supplemental Information 1 SAS code

## Additional Information and Declarations

### Data Availability

The following information was supplied regarding data availability: The NIS database raw files were purchased through online HCUP (health care cost and utilization project) distributor. All purchasers and users of HCUP data must complete the online Data Use Agreement (DUA) training so that they are familiar with the rules and restrictions for the use of HCUP data. If this raw data is made public, then the likelihood of maintaining standards to complete the DUA training might be violated. Moreover, anyone who uses the database files we purchased must complete an HCUP Data Use Agreement form (a form which the owner of the data has to generate and acknowledge). Therefore, given these limitations, we have provided raw data for review purposes and not for publishing (i.e., to be shared with the public). Additionally, as per AHRQ-HCUP, before the reviewers/editors may access any HCUP data (the raw files), they are required to complete the 15-minute Data Use Agreement (DUA) training and read and sign the HCUP DUA. Both of these may be completed online at this link: https://www.hcup-us.ahrq.gov/tech_assist/dua.jsp.
